# Cloning and enhancing lumbrokinase production from local *Eisenia fetida* by signal peptide engineering for effective thrombosis treatment

**DOI:** 10.1371/journal.pone.0328393

**Published:** 2025-07-24

**Authors:** Adeeba Tabassum, Nadia Zeeshan, Amber Afroz

**Affiliations:** Department of Biochemistry and Biotechnology, University of Gujrat, Gujrat, Punjab, Pakistan; AARI: Ayub Agricultural Research Institute, PAKISTAN

## Abstract

Thrombosis, the formation of blood clots in the circulatory system, is a major global health concern. Lumbrokinase, found in earthworms, is promising natural source of antithrombotic compounds. Traditional extraction methods from earthworms can be challenging due to contamination, making genetic engineering an attractive option. In this study lumbrokinase gene from the local earthworm species *Eisenia fetida* was isolated, sequenced, and cloned in the expression vector. The full-length lumbrokinase cDNA spanning 744 nucleotides was successfully amplified by PCR and the obtained sequence was submitted at GenBank under the accession # OP820958. The active side residues, 2D and 3D structure of the deduced protein sequence (248-amino acid) were determined by I-TASSER. Moreover, an innovative recombinant expression strategy (i.e., expression in pET22b (+) vector with *pel*B signal peptide and the pET28a (+) vector with SUMO tag) was employed to enhance lumbrokinase production and yield. Lumbrokinase amounting to approximately 6.8 mg/l in pET22 b (+) and 8.9 mg/l in pET28-SUMO was obtained from 1-liter culture. In the fibrin plate assay, specific activities of 1942 U/mg and 2027 U/mg were found from periplasmic and cytoplasmic space, respectively. In an in vitro blood clot lysis assay, recombinant lumbrokinase showed 25.67% and 66.23% clot lysis after 240-minute incubation at 37°C. This study discovered the presence of lumbrokinase genes in local earthworms and made significant contributions to the development of recombinant fibrinolytic enzymes with prospective applications in thrombotic disease treatment as an alternative to conventional approaches.

## Introduction

Thrombosis is a leading cause of death and disability worldwide. Intravascular thrombosis, the primary cause of cardiovascular disease, has a significant impact on morbidity and death accounting for I in 4 deaths globally every year. The expected increase to deaths by cardiovascular diseases is 23.3 million people by 2030 [1,2].

Traditionally, thrombosis treatment involved anti-platelet and anti-coagulating agents or surgery [3] Fibrinolytic enzymes, capable of dissolving fibrin clots, are considered potential thrombolytic agents for various cardiovascular diseases. Various sources, including plants, animals and microorganisms, yield a diverse range of fibrinolytic enzymes. Earthworm fibrinolytic enzymes (EFE) are alkaline serine proteases also known as Lumbrokinases (LK) are well-recognized for their use in the medical cure of blood clotting diseases, typically in stroke [4]. *Lumbricus rubullus (L. rubellus)* and *Eisenia fetida* (*E. fetida)* are major sources of fibrinolytic medications in Southeast Asian countries [5]. EFE is an extract of enzymes and a blend of extracts is sold under the trade names Panford or Boluoke as an oral supplement for healthy cardiovascular function [5].

In Pakistan, earthworms are abundant in soil fauna, with 42 diverse species present in the terrestrial environment [6]. Assigning earthworms for medicinal use provides cost-effective treatments for various blood clotting diseases, addressing health challenges, promoting affordability, and driving innovation in the pharmaceutical sector, thus contributing to economic growth [7].

EFE extracts prepared directly from organisms (like earthworms) face several challenges like heterogeneity, contamination, low yield, toxicity, and health risks. However, tailored heterologous expression strategies offer a viable alternative to optimize the production of these antithrombotic proteins for effective and safe clinical use [8]. Genes encoded the lumbrokinase from different species of earthworm were previously reported in different reports, only a few demonstrate fibrinolytic activity when cloned and expressed in *E. coli* typically resulting in low yields or partial activity [9–11].

Enhancing the quantity and functionality of recombinant proteins involves addressing challenges like inclusion body formation, low yield, low solubility of heterologous protein and facilitating purification. Fusion tags with distinct objectives, such as SUMO and Pel B, have been employed in numerous researches. PelB signals enhance periplasmic protein expression, folding, and stability, simplifying purification by post-osmotic shock [12–14].

On the other hand, the SUMO fusion tag enhances the expression, solubility, and stability of recombinant proteins, increasing yield by preventing degradation. It boosts expression without compromising host cell viability for proteins prone to form inclusion bodies [15].

In this study, a gene encoding lumbrokinase from locally available earthworm *E. fetida* was sequenced and cloned. Heterologous expression of isolated lumbrokinase was achieved in *E. coli* as fusion proteins by employing two strategies: pelB signal peptides in pET22b (+) vector for precise folding and SUMO tags in pET28a (+) vector for enhanced solubility and yield. Recombinant lumbrokinase showed promising fibrinolytic potential as shown by the fibrin plate method and in vitro blood clot lysis.

## Materials and methods

### Sampling

*E. fetida* were collected from locations (S1-S8) in the area of Bhimber, Azad Kashmir (Pakistan), by hand sorting method [16] from April to October. Collections were made from a variety of environments, including agriculture fields, and cow manure waste dump soil where the earthworms found most prevalent.

The mud and contaminants on the surface of the earthworms were removed by extensive washing to clean their gut and submerged in water for at least 24 hours before further processing.

### Total RNA extraction and cDNA synthesis

*E. fetida* was dissected and tissue of the pharyngeal region, crop, gizzard, and the initial segment of the intestine from the earthworm was collected for the extraction of total RNA using Trizol reagent by following the manufacturer’s instruction with some modifications described as below (Invitrogen, Carlsbad, CA, USA). The freshly extracted digestive tract tissue (100 mg) was mixed with 1000 µl of Trizol reagent and ground to a fine consistency using a cooled mortar and pestle. The homogenized sample was transferred to the Eppendorf tube by pipetting and 400 µl of phenol: chloroform mixture (1:1 ratio) was added, mixed for 10 minutes and centrifuged for 10 minutes at 11000 rpm. Following centrifugation 500 µl of isopropanol was added to the upper aqueous layer in a separate tube to precipitate out the RNA. The mixture was centrifuged at 11,000 × g for 10 minutes after being incubated for 10 minutes at room temperature. RNA pellet was suspended in 50 µl of nuclease-free water.

The cDNA was synthesized from RNA with Revert Aid First Strand cDNA Synthesis Kit (Cat #: K2563; Thermo Scientific™). The reaction mixture was prepared by pipetting 5µl of total RNA (1 µg), and 2 µl of Oligo (dT)_8_ primer in a total volume of 20 µl, all other components are the same as mentioned by the kit protocol. The final reaction was mixed, centrifuged briefly and incubated at 42°C for 60 min in a thermal cycler to carry out the cDNA synthesis.

### Gene synthesis by polymerase chain reaction (PCR) and sequencing

*E. fetida* lumbrokinase 3 precursor mRNA specific degenerate primers (using multiple sequence alignment; S2 Fig supporting data) were designed as follows: forward primer: 5’-GACCATACGAGTTCCCRTGGC-3’ and reverse primer: 5’-AGTTGTTGGTGATCR TGTC-3’ (where R stands for A/G and Y stands for G/A) and polymerase chain reaction (PCR) was carried out using 4 µl of cDNA as a template. High-fidelity taq (IU) and 2.5 mM MgCl_2_ were used for PCR amplification. used for subsequent polymerase chain reaction. cDNA was used for the first round of PCR amplification

The PCR conditions were set as follows: initial denaturation at 94°C for 3 minutes, followed by 30 cycles of denaturation (94°C for 45 sec), annealing (49.55°C for 60 sec), extension (72°C for 45 sec) and final incubation at 4°C. Amplified product was run on 1% agarose gel electrophoresis. The PCR product was purified from gel using QIAquick gel extraction kit (cat. nos. 28704) as per the recommended protocol.

The purified PCR-amplified product was cloned into the T/A cloning pTZ57R/T vector as per manufacturer’s recommended protocol (Thermo Scientific InsTAclone PCR Cloning Kit. cat #K1213). The 30 µl of ligation mixture was prepared by assembling the following components in the microfuge tube: 3 µl of pTZ57R/T (0.165 µg), purified PCR product 7 µl (330 ng), 6 µl of 5X ligation buffer, 1 µl of T_4_ DNA ligase (1U), 13 µl of nuclease-free water and mixture was incubated at 4°C for 12 hours. After incubation ligation mixture was used to transform competent *E. coli* DH5α cells by heat shock method. The resultant recombinant T/A plasmid was sequenced by dideoxy chain termination method using universal primers M13F and M13R. The sequence thus obtained was submitted to GenBank under accession number OP820958.

### *In-silico* analysis and prediction of protein structure

Computational analysis was performed to predict ORFs, translation to predict the protein sequence (http://www.dnatoprotein.com). The predicted protein sequence was submitted to the NCBI Protein database (https://www.ncbi.nlm.nih.gov/protein) for annotation and further analysis. The lumbrokinase protein sequence was submitted to I-TASSER (http://zhanglab.ccmb.med.umich.edu/I-TASSER/), an online web server for protein 3D structure designing [17]. The proposed models designed by I-TASSER were verified through Verify3D and PROCHECK (https://services.mbi.ucla.edu/SAVES/) for the suitable 3D structure.

### Cloning in expression vector

Two pair of primers Fp-LB (5*’*-GAATTCGATGGAACTTCCTCCC-3*’*), Rp-LB (5’-AAGCTTTCAGTTGTTGGTAATAATG-3’) and Fs-LB (5*’*- GAATTCATGGAACTTCCTCCCGGA-3*’*), Rs-LB (5*’*-CTCGAGTCAGTTGTTGGTAATAATGTC-3*’*) were designed for cloning in pET22b (+) and pET28a (+) SUMO respectively. Fp-LB, Rp-LB have *Eco*RI *and Hind* III and Fs-LB, Rs-Lb has *Eco*RI and *Xho*I recognition sequences respectively (shown as underlined and italicized) at 5′ and 3′ ends of the coding region. Reaction mixture was prepared for PCR amplification of lumbrokinase separately for pET22b (+) and pET28a SUMO (+) vectors using the reaction conditions as follows: initial denaturation at 94°C for 3 minutes followed by 30 cycles: denaturation (94°C for 45 sec), annealing (at 48°C for Fp-Lb, Rp-LB and at 53.6°C for Fs-Lb, Rs-LB for 60 sec), extension (72°C for 45 sec) and final incubation at 4^0^C. The PCR products were double digested by their respective restriction enzymes. Ligation was performed at 4°C (+) for 12 hours by incubating the double digested vector (pET22b and pET28a SUMO) vector and their respective restricted PCR products with T4 DNA ligase. Chimeric pET22b-LB and pET28SUMO-LB expression plasmids were maintained in *E. coli* DH5α, selected on LB medium containing 100 μg/ml ampicillin and 50 μg/ml kanamycin respectively. The junction sequences of the recombinant plasmid were confirmed by restriction analysis and colony PCR.

### Expression and purification of recombinant lumbrokinase

For expression analysis, transformed *E. coli* BL21 (DE3) was grown overnight in 50 ml LB-ampicillin/kanamycin medium at 37°C. Fresh LB medium (250 ml) was inoculated with overnight culture (1%) followed by incubation at 37°C in an orbital incubator shaker at 150 rpm. Lumbrokinase expression was induced with 1 mM isopropyl-β-d-1-thiogalactopyranoside (IPTG) at a cell density of A_600_ of 0.8. Cells were allowed to grow till the maximum growth was attained (~ 12 h) and then harvested by centrifugation at 6500 rpm for 15 min. During incubation, 1 ml aliquots were collected every 2 h to monitor the growth (A_600_) and analyze protein expression by 12% SDS-PAGE. The percentage of lumbrokinase expression in total cell proteins was determined by densitometry of the Coomassie stained gel. For purification and recovery of periplasmic and cytoplasmic lumbrokinase one liter induced culture of transformed *E. coli* BL21 (DE3) harboring pET22b-LB and pET28SUMO-LB were centrifuges at 6500 rpm for 15 minutes at 4°C and resulting pellet was used for further processing.

### Recovery of periplasmic lumbrokinase

**Fig 1:**
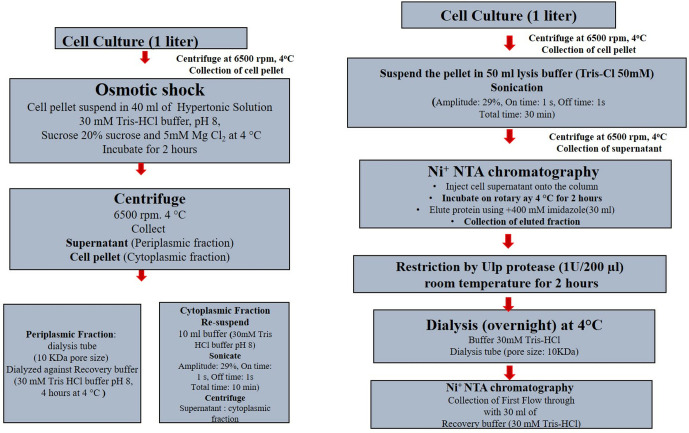
Purification of recombinant lumbrokinase. (A) periplasmic extraction (B) cytoplasmic extraction.

For the extraction of lumbrokinase from periplasmic space, cells were washed by resuspending it in 400 ml of ice cold washing buffer (30 mM Tris-Cl pH 8.0) and then centrifuging at 6500 rpm for 15 minutes at 4°C. The resulting washed cell pellet was suspended in 40 ml of ice cold hypertonic buffer (30 mM Tris-Cl pH 8.0, 20% sucrose, 5 mM MgSO_4_) and incubated on ice for two hours. After centrifugation, the supernatant was dialyzed against 1 liter of recovery buffer (30 mM Tris-Cl, pH 8.0) for 4 hours with two time changes at 4°C and pooled sample were collected for subsequent activity assays as outlined in Fig 1A [18].

### Recovery of cytoplasmic lumbrokinase

Induced culture of *E. coli* (BL21DE3) harboring the pET-SUMO LB was grown in 500 ml LB media was harvested by centrifugation (6500 rpm, 15 min), resuspended in 50 ml of 30 mM Tris-Cl buffer (pH 8.0) and lyzed by sonication (10 × 40 s bursts with intervals of 30 Sec between successive pulses) in a UP 400s Ultrasonicator (Dr. Hielscher GmbH, Germany). Lysate thus obtained was again centrifuged as described previously, and soluble protein in the supernatant was clarified by passing through a syringe filter (0.45 µM) and loaded Ni-NTA agarose beads (Qiagen) pre-equilibrated with buffer W (10 mM Tris-Cl 1 mM NaCl) incubated on a shaker for 1 hour at 4 C. Following the incubation period, the column was twice washed: once with 40 ml of buffer W to elute the unbound protein, and again with 20 ml of buffer W supplemented with 200 mM imidazole. In protein sample 1 U of Ulp protease were added and further incubated for 12 hours at 4°C in order to remove the SUMO tag. The pre-equilibrated Ni-NTA agarose beads column was once again loaded with cleaved protein samples and the SUMO tag-cleaved protein was now collected by washing the column with 20 ml of W buffer as unbound protein. Finally, the column was washed with 1 M imidazole to remove SUMO-tag and then with excess deionized water for reuse as outlined in Fig 1B.

### Fibrinolytic activity assay for lumbrokinase

Lumbrokinase fibrinolytic activity was measured using an improved fibrin plate method as described by Stephani [19]. Fibrin plates were prepared by adding 1.0% agarose in PBS buffer (1X) supplemented with thrombin (IU/ml) and fibrinogen (1 mg/ml) in a petri dish. 100 μl of protein samples (0.5 mg protein/ml) was poured into a 5 mm sterile filter paper disc and incubated overnight at 37°C. Measure clear zone diameters around each disc with a ruler, calculating mean values from three replicates (n = 3). The activity of samples was calculated as the lytic area adjacent to the disc on the fibrin plate relative to standard lumbrokinase.

### Blood-clot lysis activity assay and statistical analysis

An *in vitro* experiment assessed blood-clot lysis activity was measured by following the protocol described by Prasad [20]. Initially, 500 μl of fresh blood was added to pre-weighed 1.5 ml Eppendorf tubes and incubated at 37°C for 2 hours. After clot formation, serum was removed, and clot weight was determined. Then, 200 μl of standard lumbrokinase solution of 99% UP (2000 U/mg) and 200 µl of test lumbrokinase with varying doses (0.5, 1.0, 1.5, 2.0 mg/mL) of the fibrinolytic agent in 1x PBS were added to the tubes and incubate the samples at 37°C. The percentage of clot lysis, assessed by weighing before and after incubation, yielded mean results with standard deviation (n = 3), and significant differences were determined using a t-test (p < 0.01).

The EC_50_ was determined using the AAT Bioquest EC50 Calculator. Calculated via a four-parameter log-logistic regression model (AAT Bioquest, Inc., Pleasanton, CA, USA; Quest Graph IC_50_ Calculator) available at https://www.aatbio.com/tools/ec50-calculator.

## Results

### Earthworm sample collection and identification

**Fig 2:**
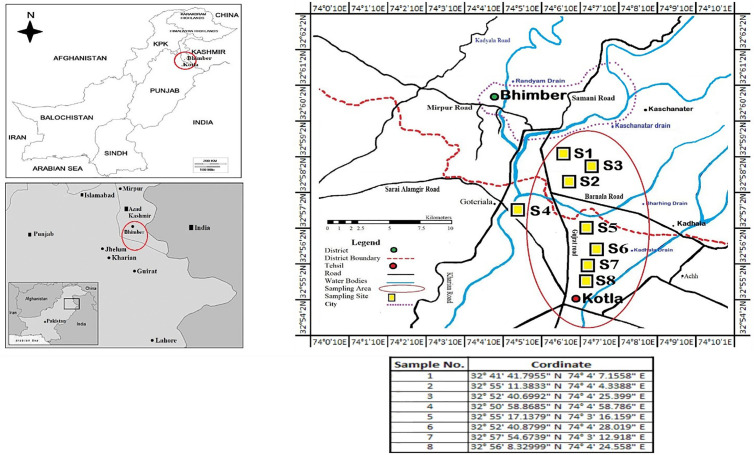
Map showing the sample collection site with coordinate generated by AutoCAD 2006.

**Fig 3:**
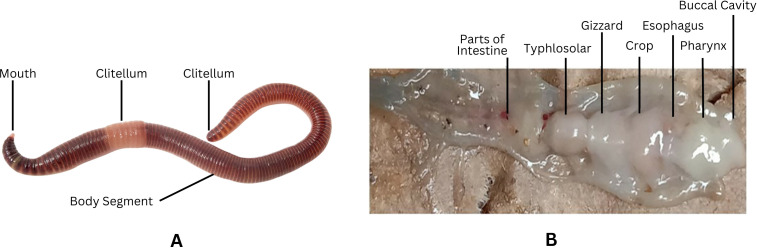
Morphological characteristics of locally isolated *E. fetida.* (A) Morphology of the collected *E. fetida* (B) Dissected *E. fetida* showing internal visceral organ.

The sample was collected from various sites in Azad Jammu and Kashmir, and identification of the earthworm was done using keys from Stephenson (1923) as described elsewhere (Julka, Blanchart et al., 2004) (Fig 2) [21]. The earthworm species *Eisenia fetida* was characterized by distinct morphological features, including a tubular, reddish-brown body composed of approximately 115 segments. Its foremost body segment, the prostomium, exhibited an epilobous form, while a saddle-shaped clitellum was positioned over segment 25. Domínguez et al. (2005) documented these attributes [22], noting that the species measured around 120 mm in length and 5 mm in diameter. A key anatomical feature was the first dorsal pore, located between segments 4/5 and 5/6. Additionally*, E. fetida* possessed two pairs of seminal vesicles, situated in the 11th and 12th segments. Its dorsal side displayed a darker coloration compared to the paler ventral side, further distinguishing its appearance (Fig 3, Table 1). Given these defining characteristics*, E. fetida* was conclusively selected for the dissection and their gut was used for RNA preparation. Moreover, the lumbrokinase extracted from these earthworm species was characterized in our previous studies as described elsewhere [23].

**Table 1:** Characteristics of Collected *E. fetida.*

Habitat	Body shape/ color	Number of segments	Length (L × W) mm	Anatomy	1^st^ dorsal pore	Vesical seminal	Identified AS
manure worm thrive in rotting vegetation, manure	Tubular Reddish brown	~ 115 ± 2	120 ± 1	Clitellum start from segments 25	between segments 4/5, 5/6	2 pairs, in 11^th^ and 12^th^ segment	*Eisenia fetida as described by* Julka (2004) [21]

### Cloning and sequencing of lumbrokinase

**Fig 4:**
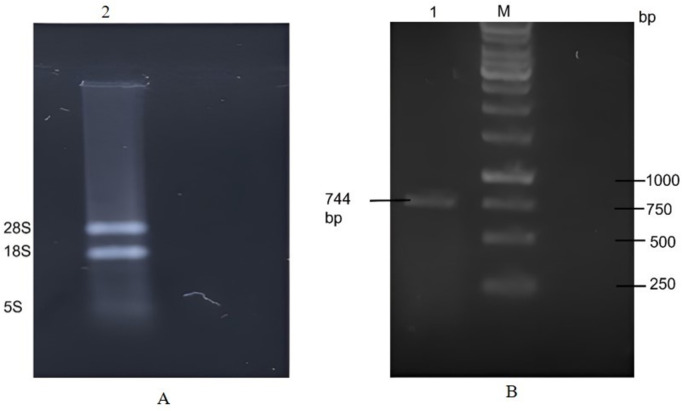
1% agarose gel electrophoresis. (A) Lane 2 represents total RNA extracted from earthworm. (B) PCR amplification product with LKF, LKR primers, Lane M; 1Kb DNA ladder, Lane 1: amplified PCR product.

**Fig 5:**
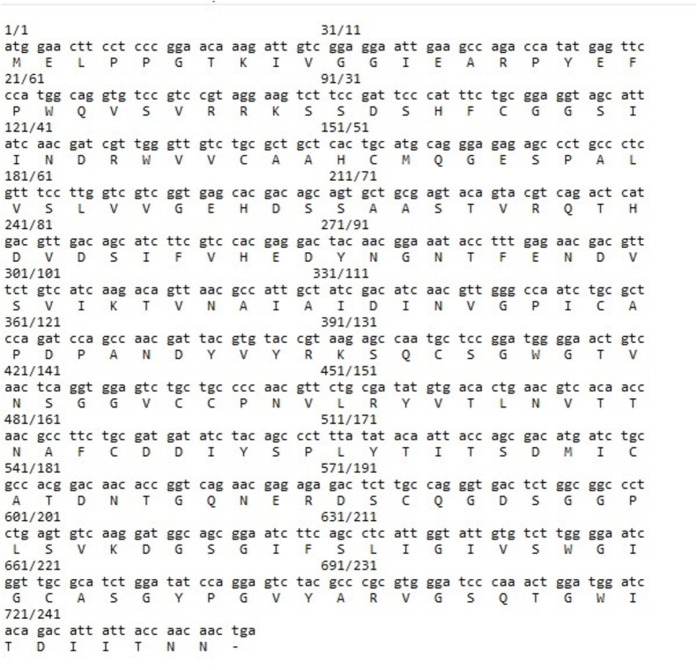
Lumbrokinase nucleotide (upper line) obtained from sequencing and deduced amino acid (lower line) sequences. (GenBank: OP820958; Protein ID: WGV41589).

The lumbrokinase total cDNA fragment was synthesized through RT-PCR, utilizing *E. fetida* total RNA as a template (Fig 4A). The DNA fragment encoding the lumbrokinase gene was amplified by using the specific primers LKF and LKR after 30 cycles of reaction (Fig 4B). The amplified PCR fragment was sequenced and the nucleotide sequence and the corresponding deduced amino acid sequence of lumbrokinase have been submitted to GenBank under accession numbers GenBank: OP820958; Protein ID: WGV41589. The lumbrokinase cDNA comprises 744 nucleotides, and the analysis of the deduced amino acid sequence within the open reading frame, spanning from 1 to 744, reveals a 258-amino acid protein with a molecular weight of 27.17 kDa (Fig 5).

### Prediction of lumbrokinase 2D and 3D structure

**Fig 6:**
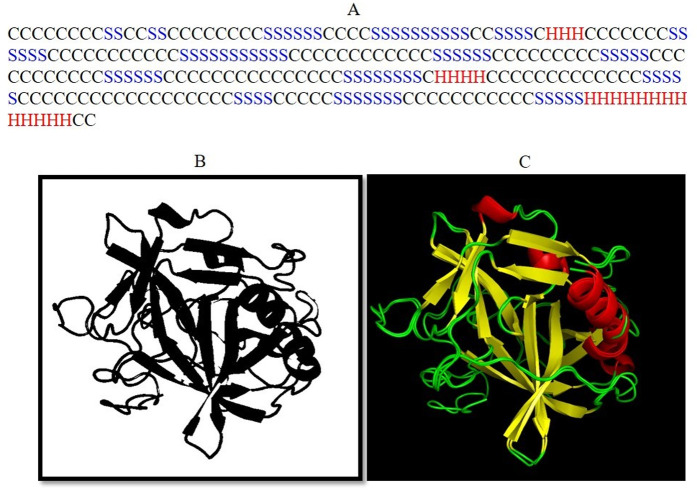
Structural characteristics of lumbrokinase. **(A)** The 2D structure prediction lumbrokinase (C represents the coils, H represents the helix and S represents the β-pleated sheets); **(B)** 3D model of lumbrokinase predicted by I-TASSER **(C)** Superimposed structure of lumbrokinase and its structural analogue1YM0A.

From the obtained protein data, sequence-based secondary structure was predicted by I-TASSER includes coil (C) approximately 31.70%, β-sheet (S) comprises approximately 20.09% of the sequence and α-helix (H) comprises approximately 4.91% of the sequence (Fig 6A).

3D structure was predicted by I-TASSER web server that can generate high-quality PDB models. The obtained top I-TASSER model has a C-score of 0.47, with an estimated TM-score of 0.78 ± 0.10 and an estimated RMSD of 4.8 ± 3.1Å, as shown in the (Fig 6B). TASSER modeling selects the template 1YM0A with the highest Z-score (4.33), representing the Crystal Structure of earthworm fibrinolytic enzyme component B, an Innovative Glycosylated Two-chained Trypsin from the PDB collection by LOMETS. COACH predicted binding sites at positions 51, 96, 172–195, 197, 216–222, and 228. The ligand type is identified as 0G6, a peptide according to BioLIP2. COFACTOR predicted function, suggesting EC number 3.4.21.117 with a confidence score of 0.653. It’s associated with serine protease 6, known for cleaving proteins with aromatic side chains at position P1, belonging to peptidase family S1A, with active sites at residues 51, 99, 193–196.

### Construction of expression vector of lumbrokinase

**Fig 7:**
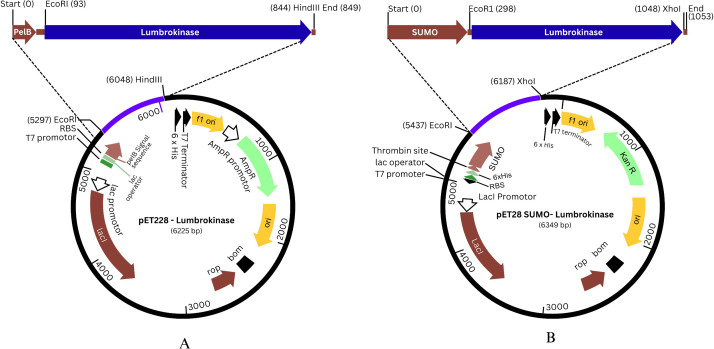
Heterologous expression vector. (A) recombinant pET22b-lumbrokinase (B) recombinant pET28-SUMO –lumbrokinase.

**Fig 8:**
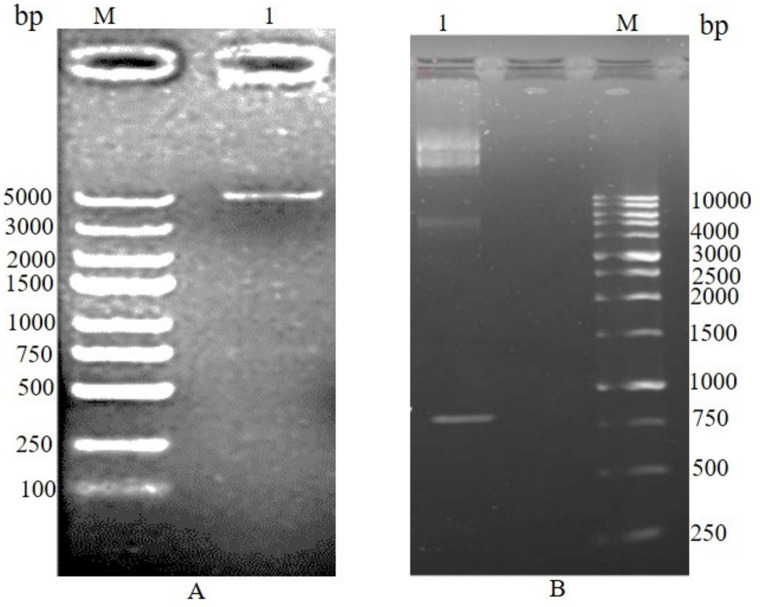
Agarose gel electrophoresis of restriction analysis of recombinant vectors. (A) pET22b (+) harboring lumbrokinase restricted with EcoR1 and HindIII; (B) pET28a SUMO harboring lumbrokinase restricted with Eco*R*1 and XhoI. Lane M: 1 kb DNA ladder; Lane 1: Amplified PCR product.

About 744 nucleotide long DNA fragments of lumbrokinase were amplified by Polymerase chain reaction with the two sets of primers containing *Eco*RI and *Hind*III sites in LKF22/LKR22 primers and *Eco*RI and *Xho*I sites in LKF28/LKR28 primers at the 5′ and 3′ termini, respectively. The DNA amplified by LKF22/LKR22 pair was cloned in T7/*lac* promoter-based pET-22b (+) vector at *Eco*RI/ *Hind*III sites to generate the pET22(b)-pelB lumbrokinase recombinant vector and DNA amplified by LKF28/LKR28 pair was cloned in T7/*lac* promoter-based pET-28a (+) vector at *Eco*RI/ *Xho*I sites to generate pET- 28(+) SUMO lumbrokinase recombinant vector (Fig 7 A, B). Initially, both recombinant plasmids were maintained in *E. coli* DH5α for stable vector propagation and then transformed into *E. coli* BL21 (DE3) Codon Plus for expression studies. The presence of the lumbrokinase gene (744 bp) was confirmed by colony PCR (S1 Fig) and restriction digestion as shown in (Fig 8). The use of two restriction sites at 5’ and 3’ of the coding regions enabled the successful in-frame insertion of genes as reported previously [24,25].

### Heterologous expression of lumbrokinase

**Fig 9:**
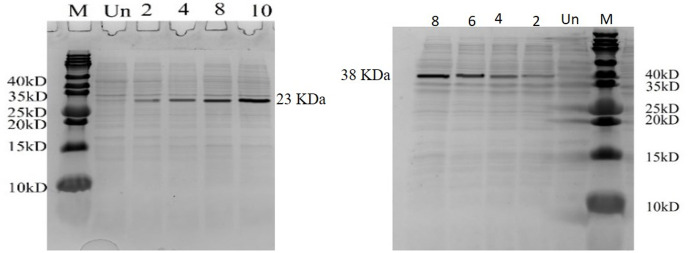
SDS-PAGE analysis of total cell proteins from *E. coli* BL21 (DE3). (A) harbouring pET22b (+)-lumbrokinase: Lane 1, uninduced sample; Lanes 2-5, samples induced with 1 mM IPTG at 28ºC for 2, 4, 8, and 10 hours, respectively; M, protein molecular weight marker. (B) harbouring pET28a (+) SUMO-lumbrokinase: Lane 1, uninduced sample; Lanes 2-5, samples induced with 1 mM IPTG at 37ºC for 2, 4, 6, and 8 hours, respectively; M, protein molecular weight marker.

*E. coli* cells harboring recombinant pET22b (+) and pET28a (+) were grown in LB-ampicillin and LB-Kanamycin, respectively were induced with 1mM IPTG. Total cell protein analysis of the induced *E. coli* cells by SDS-PAGE showed a band at a position corresponding to ~ 38.47 kDa and ~29.37 kDa, which matches the calculated molecular mass of Lumbrokinase with SUMO and pelB fusion tags. The maximum expression levels were found to be ~ 10% and 16% for pelB and SUMO tag respectively, within ~ 10 h and 8 hours of induction and remained constant thereafter (Fig 9).

### Purification of lumbrokinase

**Fig 10:**
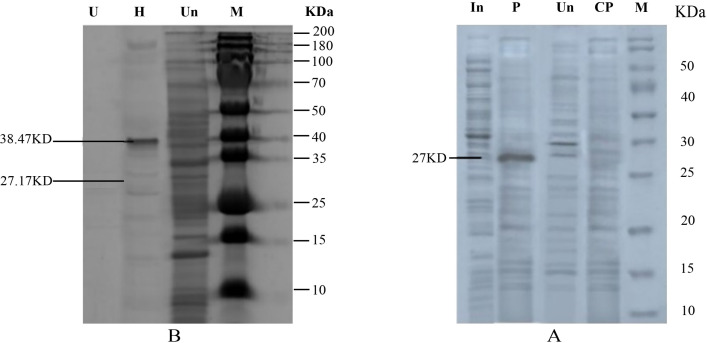
SDS-PAGE of lumbrokinase at different stages of purification. (A) expressed with pelB signal: CP, cytoplasmic protein; Un, uninduced sample; P, Periplasmic; (B) expression with SUOMO tag: In, induced sample; Un, un induced sample, H, His taq purified; U, final product after Ulp protease treatment; M, Protein marker.

After optimization of expression, the lumbrokinase was recovered from the periplasmic space of transformed *E. coli* BL21(DE3) cells harboring pET22 (+) by using the osmotic shock resulting in successful extraction and purification of the 6.8 mg of lumbrokinase with 18.37% recovery from 1000 ml of bacterial culture (Fig 10, Table 2).

**Table 2:** Comparison of activities of lumbrokinase recovered from periplasmic space (expressed in pET-22b) and cytoplasmic space (pET28-a SUMO) from 1liter culture.

Plasmid	Location	Total Cell Proteins* (mg)	Percentage of Expression (%)	Total Lumbrokinase (mg)	Purified Lumbrokinase (mg)	Lumbrokinase Activity (U)	Specific Activity (U/mg)	Recovery (%)	Purity** (%)
pET22b (+)	Periplasmic	370	10	37	6.8	13,200	1942	18.37	71.5
pET28a(+)SUMO	Cytoplasmic	540	16	86.4	22.5	48840	2170	20.921	92

*Total cell proteins means the protein concentration obtained after sonication of cells.

**purity was calculated by divide the background-corrected density of the protein band on SDS-PAGE by the background-corrected density of the whole lane and multiply by 100. (There is an assumption here that all proteins stain equally well).

The purification of recombinant lumbrokinase from *E. coli* BL21 (DE3) harboring pET-28a (+) involved the removal of fusion tags (SUMO), to obtain the native form of the enzyme. For the effective purification of target lumbrokinase, we used Ni-NTA affinity chromatography column, (Qiagen, Hilden, Germany) by immobilization of the lumbrokinase twice, both before and after Ulp protease treatment. In the first immobilization, lumbrokinase was eluted with 40% imidazole and laterally, lumbrokinase was eluted as unbound protein. After purification, lumbrokinase showed 92% purity purification with recovery of 20.921% from 1000 ml culture (Fig 10, Table 2). Our findings suggested that the production and purity of lumbrokinase expressed with SUMO tag was 1.25-fold and 1.35 fold higher respectively than that recovered from periplasmic space.

### In-vitro activity analysis

#### Fibrin plate assay.

**Fig 11:**
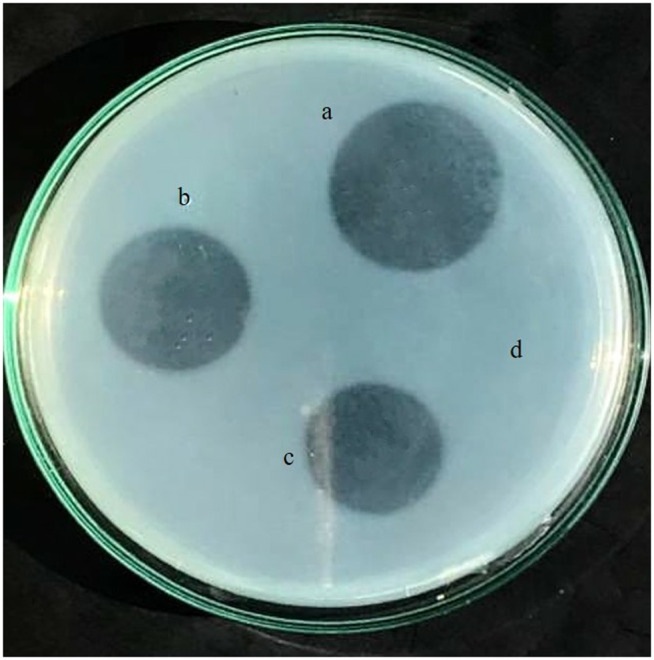
Fibrin plate assay a: Positive control (standard lumbrokinase), b: purified cytoplasmic recombinant lumbrokinase pET-28a (+) SUMO, c: Purified periplasmic recombinant lumbrokinase pET-22b (+), d: Negative control (1 × PBS buffer).

The activity of recombinant lumbrokinase was assessed by determining the hydrolysis region on the fibrin plate. Both fractions recovered from periplasmic space and purified from cytoplasm showed a clear zone of hydrolysis suggesting their fibrinolytic activity (Fig 11). The fibrinolytic activity of both fractions was quantified based on the diameter or area of the hydrolysis zones observed on the fibrin plates. The fibrinolytic activity of periplasmic lumbrokinase and purified cytoplasmic lumbrokinase was determined to be 1942 U/mg and 2170 U/mg, respectively, utilizing the standard lumbrokinase curve for measurement.

#### In vitro blood-clot lysis assay and calculation of EC50.

**Fig 12:**
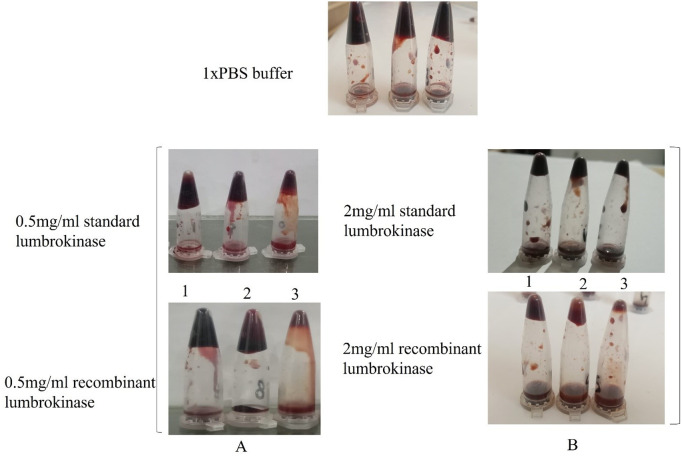
Dose-time dependent blood clot lysis: recombinant lumbrokinase and standard lumbrokinase. **1X PBS Buffer negative control (2, 4, and 6 hours of incubation).** (A) 0.5 mg/ml standard and recombinant lumbrokinase, tubes 1, 2, and 3 after 2, 4, and 6 hours of incubation, respectively. (B) 2.0 mg/ml standard and recombinant lumbrokinase, tubes 1, 2, and 3 after 2, 4, and 6 hours of incubation respectively.

**Fig 13:**
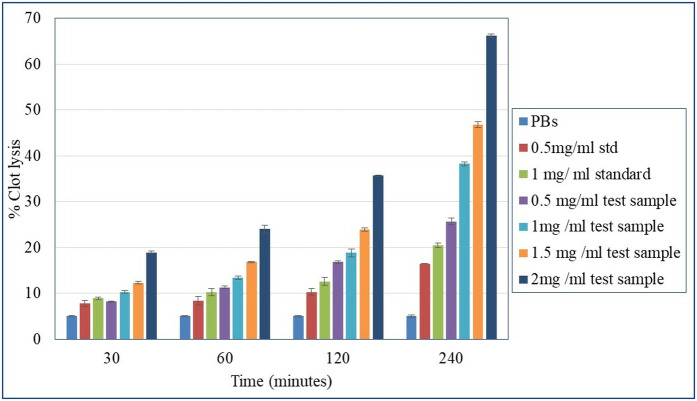
Percentage clot lysis activity: Dose-time dependent blood clot lysis of lumbrokinase.

The blood clot hydrolysis assay was conducted to evaluate the fibrinolytic activity of recombinant lumbrokinase. The extent of fibrinolysis was quantified by calculating the percentage of clot dissolution or the reduction in clot weight or volume relative to the initial clot size (Figs 12 and 13) at different doses (0.5–2 mg/ml) and incubation times (30–240 minutes). The lumbrokinase at concentration of 2 mg/ml was found to dissolve 70–80% of the clot after 240 minutes of incubation shows its effectiveness (S1 and S2 Tables).

**Fig 14:**
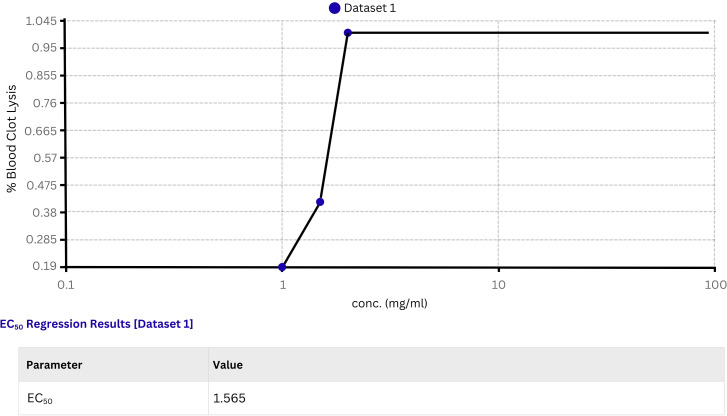
Graph represents the EC50 of recombinant lumbrokinase calculated by AAT Bioquest EC50 calculator.

The EC50 is an important parameter for assessing efficacy, especially in fibrinolytic enzymes for thrombolysis and a value of 1.565 mg/ml for the fibrinolytic enzyme indicates potent efficacy, requiring a low concentration for effective thrombolysis (Fig 14). This underscores its clinical relevance in treating thrombotic disorders, crucial for rapid and efficient clot dissolution.

## Discussion

The therapeutic potential of earthworm fibrinolytic enzymes (EFE) in the management of blood clotting disorders has been garnered a great deal of attention. However heterologous expression in e. coli remained a changed due to numerous obstacles including poor or negligible production and the formation of inclusion bodies as a result of low solubility. In this study, efforts have been undertaken to boost the production of active lumbrokinase from *E. fetida* by employing two key strategies: periplasmic secretion using a pelB signal sequence and solubility enhancement via SUMO fusion.

Different lumbrokinase genes designated as PV 242, PI 239, PM 246, F6, EFE-3, CST1, F 238 mostly spanning ~900 bp length was cloned and sequence from different earthworm species [9,26–29]. In this study, a full-length lumbrokinase cDNA, spanning 744 nucleotides and encoding a 248-amino acid polypeptide was successfully isolated from local *E. fetida* earthworms. Our predicted secondary structure of cloned lumbrokinase aligned well with previous reports, exhibiting 31.70% random coils, 20.09% β-sheets, and 4.91% α-helices, consistent with 2D structure characteristics of previously reported isozymes such as EFE-a, EFE-b, Ef P-0, Ef P-I, Ef P-II, and Ef P-III [30].

The catalytic properties of earthworm-derived fibrinolytic enzymes are closely tied to their three-dimensional structure. The crystal structure of EFE‑B—resolved under PDB entry 1YM0A (Z-score = 4.33) revealed a glycosylated, two-chain serine protease adopting a classic trypsin fold. Notably, 1YM0A corresponds to a clinically used oral thrombolytic agent in East Asia, known for its stability and efficacy in treating thrombosis [31]. *In-silico* superimposition of our recombinant lumbrokinase onto the 1YM0A structure showed substantial backbone and active-site alignment, particularly around the catalytic triad (His-Asp-Ser) and substrate-binding clefts (Fig 6c). This structural congruence strongly implies shared enzymatic mechanisms and substrate specificity between cloned recombinant lumbrokinase and the native earthworm enzyme.

Li *et al.* (2008) reported the expression of lumbrokinases (lrF1 and lrF2) from *Lumbricus rubellus* using the pET28a(+) vector in *Escherichia coli* BL21 (DE3). The recombinant proteins (lrF1 and lrF2) accounted for approximately 40% of total cellular proteins; however, they mostly accumulated as insoluble inclusion bodies, necessitating refolding steps to restore fibrinolytic activity [32]. Similarly, another study reported that approximately 10% of the total protein, corresponding to inclusion bodies of lumbrokinase PI239 from *Lumbricus bimastus*, are recovered from in vitro refolding to obtain functional protein [33]. In contrast, our study demonstrated heterologous expression of lumbrokinase with yields of 10% and 16% of total cellular proteins in *Escherichia coli* BL21 (DE3) when fused with pelB and SUMO tags, respectively. Notably, in both cases, the lumbrokinase was mostly expressed in a soluble form, eliminating the need for refolding and thereby simplifying downstream processing.

Previously another expression system *Pichia pastoris* also proved effective for earthworm fibrinolytic enzymes (EFEs), offering variability among different EFEs with higher secretion levels and glycosylation potential [24,28]. However, *P. pastoris* systems typically yield in the range of 10–30 mg/l for secreted enzymes, often requiring longer induction periods [34]. In contrast, *E. coli* offers a faster, cost-effective system where the pel B SUMO-tagged lumbrokinase achieved 6.8 and 22,5 mg/l with high solubility and bioactivity within 6 hours of induction. This strategy enhanced soluble expression and amplifies the production and efficacy of growth hormone as reported by Clark, (2001) [35]. Choi and Lee (2004) [36] also discussed the use of different signal peptides and their unique characteristics during recombinant protein production and their effect on secretion. Other Previous studies also reported the addition of the 22-amino-acid pelA from *Erwinia carotovora* CE attached to proteins facilitated their transfer to the periplasmic space [37].

In our study, we incorporated a 66 bp-long pelB signal sequence to our lumbrokinase enzyme (pET22b-Lumbro) which directs the secretion of recombinant proteins to the periplasmic space, yielding soluble lumbrokinase periplasmic protein amounting approximately 6.8 mg/l after 6 hours of induction at 28°C, showing specific activity of 1924 U/mg. This approach eliminated the need for extra purification steps or refolding, addressing the compromised fibrinolytic activity associated with cytoplasmic insoluble inclusion bodies [38]. Different studies reported the addition of the pelB signal sequence increases the translocation efficiency for penicillin G acylase compared to other signal peptides (OmpA, Lpp, PhoA, and MalE). Proteins such as pancreatic prokallikrein, human epidermal growth factor (hEGF), and protease inhibitors were synthesized in the periplasmic space of *E. coli* using secretion systems [39–41].

The second approach used in this work involved, attaching the small ubiquitin-like modifier (SUMO) protein to the N-terminus of lumbrokinase, which improved solubility and expression [42]. Peroutka III *et al*., 2011) reported the use of SUMO fusion for enhanced protein expression and purification in prokaryotes and eukaryotes. In another previous study, optimizing the human Hepcidin25 gene, fusion with SUMO enhanced expression, peaking at 6 hours, consistent with our findings using IPTG induction [43,44]. Another study reported the 30% of total bacterial protein expressed was achieved using SUMO fusion. Human SUMO1 and SUMO2, used as gene fusion tools, effectively amplify expression, solubility, and purification of heterologous proteins [45]. SUMO fusion with challenging proteins, like rabies glycoprotein, elevated the expression and enhances solubility in *E. coli*. The His6-SUMO-HV1 fusion gene was efficiently expressed in *E. coli* BL21, constituting more than 35% of the soluble fraction [46].

In the pET28(a)-SUMO framework, soluble lumbrokinase measured 8.9 mg/l after 6 hours of induction at 37°C, show casing the effectiveness of SUMO fusion technology. The activity assay evaluated the efficacy of recovered lumbrokinase from both periplasmic and soluble cytoplasmic fractions. Periplasmic lumbrokinase demonstrated a specific activity of 1942 U/mg on the fibrin plate, whereas cytoplasmic protein, fused with a SUMO tag, exhibited superior activity at 2027 ATU/mg. This underscores the effectiveness of SUMO tags in facilitating the expression of soluble proteins.

To the best of our knowledge, this study represents the first successful report of heterologous expression of earthworm-derived lumbrokinase using a small ubiquitin-like modifier (SUMO) fusion system in *Escherichia coli.* The recombinant lumbrokinase, expressed using the pET28a SUMO vector was efficiently purified via nickel-nitrilotriacetic acid (Ni-NTA) affinity chromatography. This construct yielded a soluble protein exhibiting a specific fibrinolytic activity of 2027 ATU/mg, which significantly exceeds the activity of CST1 (912 U/mg) previously reported from inclusion body-derived lumbrokinase [9].

In contrast, expression of lumbrokinase using the pET22b (+) vector with a pelB signal peptide yielded a slightly lower activity (1942 U/mg), although the protein was also obtained in soluble form. The enhanced activity and solubility observed with the SUMO fusion construct underscore the effectiveness of the SUMO tag in improving protein folding and stability during cytoplasmic expression in *E. coli*. These findings collectively highlight the potential of the SUMO fusion strategy to increase the yield, solubility, and functional activity of therapeutic fibrinolytic enzymes. Consequently, this approach may serve as a promising platform for the cost-effective production of recombinant lumbrokinase for pharmaceutical applications in the prevention and treatment of thrombotic disorders.

## Conclusion

In this study, we successfully cloned and heterologously expressed the lumbrokinase gene from *Eisenia fetida* in *Escherichia coli* using two distinct expression strategies: periplasmic targeting via the pelB signal sequence and cytoplasmic expression enhanced by SUMO fusion. Both approaches yielded soluble and biologically active forms of lumbrokinase; however, the SUMO fusion system showed best performance in terms of protein yield and fibrinolytic activity. The SUMO-tagged construct achieved higher solubility (2170 U/mg specific activity) and expression levels (16%) without the formation of inclusion bodies, eliminating the need for refolding and simplifying downstream processing. Functional validation through activity assays confirmed the biological efficacy of the expressed proteins, affirming their therapeutic potential. Importantly, this is the first report of utilizing SUMO fusion technology for the expression of earthworm-derived lumbrokinase, setting a precedent for its application in the production of other difficult-to-express proteins. Overall, our findings demonstrate the utility of the SUMO fusion system as a powerful tool to enhance recombinant protein expression in *E. coli*. This strategy not only facilitates the production of functionally active lumbrokinase but also holds significant promise for scalable biotechnological applications, including the cost-effective manufacturing of thrombolytic agents and other industrially or medically relevant proteins.

## Supporting information

S1 Fig Colony PCR, positive clone positive clone of lumbrokinase pET22 b (+). Colony PCR, positive clone of lumbrokinase SUMO pET28 a (+).
(PDF)

S2 Fig Multiple sequence alignment by clustal W. (PDF)

S1 Table Weight of clot after dissolution by incubating for different time intervals. (PDF)

S2 Table Percentage clot lysis activity after different time of incubation. (PDF)
