# Supplementary material for: Cloning and enhancing lumbrokinase production from local Eisenia fetida by signal peptide engineering for effective thrombosis treatment
Source: PLoS One. 2025 Jul 24;20(7):e0328393. doi: 10.1371/journal.pone.0328393 (PMC12288994; doi:10.1371/journal.pone.0328393)
Supplement: S1 Fig — Colony PCR, positive clone of lumbrokinase SUMO pET28 a (+). (PDF) [file pone.0328393.s001.pdf]

**Supporting Data**

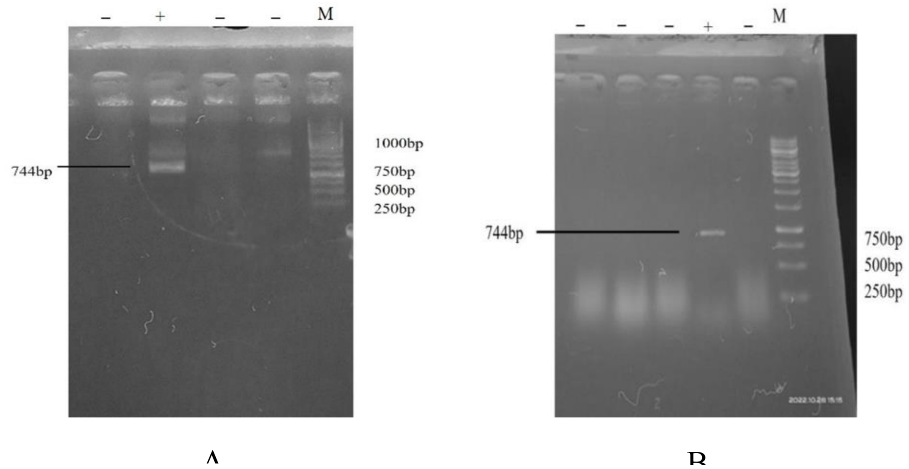


**S1 Fig: 1%** agarose gel electrophoresis of colony PCR product showed the confirmation of recombinant *E. coli* DH5ά (A) harboring pET22b-Lumbrokinase. +ve sign showed the presence of particular recombinant plasmid and –ve sign showed the absence of recombinant
