# Supplementary material for: Cloning and enhancing lumbrokinase production from local Eisenia fetida by signal peptide engineering for effective thrombosis treatment
Source: PLoS One. 2025 Jul 24;20(7):e0328393. doi: 10.1371/journal.pone.0328393 (PMC12288994; doi:10.1371/journal.pone.0328393)
Supplement: S2 Fig — (PDF) [file pone.0328393.s002.pdf]

<u>S2 Supporting Data</u>

Fig S2: CLUSTAL 2.1 multiple sequence alignment of lumbrokinase of *E. fetida* along with Accession numbers used for degenerate Primers

```
AB045720.1_1-845      --GTTACTTCTCGCTCTTGCATCGCTCGTAGCGGTGGGCTTTGCCCAACCACCAGTCTGG
U25648.1_26-872       ATGTTACTTCTCGCTCTTGCATCGCTCGTAGCGGTGGGCTTTGCCCAACCACCAATCTGG
AY438622.1_1-852      ATGTTACTTCTCGCCCTTGCATCGTTGGTAGCGGTGGGTTTTGCGCAACCACCAGTCTGG
EF545135.1_1-718      ------------------------------------------------------------
U25643.2_20-869       ATGTTACTTCTCGCCCTTGCATCGTTGGTAGCGGTGGGTTTTGCGCAACCACCAGTCTGG
AF304199.1_1-850      ATGTTACTTCTCGCCCTTGCATCGTTGGTAGCGGTGGGTTTTGCGCAACCACCAGTCTGG
U25644.1_1-718        ------------------------------------------------------------
AF433650.1_1-850      ATGTTACTTCTCGCTCTTGCATCGTTGGTAGCGGTGGGCTTTGCGCAACCACCAGTCTGG
AY684712.1_1-845      --GTTACTTCTCGCTCTTGCATCGCTGGTAGCGGTGGGCTTTGCCCAACCACCAGTCTGG
AY684711.1_1-842      --GTTACTTCTCGCTCTTGCATCGCTGGTAGCGGTGGGCTTTGCCCAACCACCAGTCTGG


AB045720.1_1-845      TACCCCGGTGGTCAATGCGGTGTCAGCCAGTACTCAGATGCTGGTGACATGGAACTTCCT
U25648.1_26-872       TACCCCGGTGGTCAATGCGGTCTCAGCCAGTACTCAGATGCTGGTGACATGGAACTTCCT
AY438622.1_1-852      TACCCCGGTGGTCAATGCGGTGTCAGCCAGTACTCAGATGCTGGTGACATGGAACTTCCT
EF545135.1_1-718      ------------------------------------------------------------
U25643.2_20-869       TACCCCGGTGGTCAATGCAGTGTCAGCCAGTACTCAGATGCTGGTGACATGGAACTTCCT
AF304199.1_1-850      TACCCCGGTGGTCAATGCAGTGTCAGCCAGTACTCAGATGCTGGTGACATGGAACTTCCT
U25644.1_1-718        ------------------------------------------------------------
AF433650.1_1-850      TACCCCGGTGGTCAATGCGGTGTCAGCCAGTACTCAGATGCTGGTGACATGGAACTTCCT
AY684712.1_1-845      TACCCCGGTGGTCAATGCGGTGTCAGCCAGTATTCAGATGCTGGCGACATGGAACTTCCT
AY684711.1_1-842      TACCCCGGTGGTCAATGCGGTGTCAGCCAGTATTCAGATGCTGGCGACATGGAACTTCCT


AB045720.1_1-845      CCCGGAACAAAAATTGTCGGAGGAATTGAAGCCAGACCATACGAGTTCCCATGGCAGGTG
U25648.1_26-872       CCCGGAACAAAAATTGTCGGAGGAATTGAAGCTAGACCATACGAGTTCCCGTGGCAGGTG
AY438622.1_1-852      CCCGGAACAAAAATTGTCGGAGGAATTGAAGCCAGACCATACGAGTTCCCATGGCAGGTG
EF545135.1_1-718      ------------ATTGTCGGAGGAATTGAAGCCAGACCATACGAGTTCCCATGGCAGGTG
U25643.2_20-869       CCCGGAACAAAAATTGTCGGAGGAATTGAAGCCAGACCATACGAGTTCCCATGGCAGGTG
AF304199.1_1-850      CCCGGAACAAAAATTGTCGGAGGAATTGAAGCCAGACCATACGAGTTCCCATGGCAGGTG
U25644.1_1-718        ------------ATCGTTGGTGGTATCGAAGCTAGACCATACGAGTTCCCGTGGCAGGTG
AF433650.1_1-850      CCCGGAACAAAAATTGTCGGAGGAATTGAAGCCAGACCATACGAGTTCCCATGGCAGGTG
AY684712.1_1-845      CCCGGAACAAAAATTGTCGGAGGAATTGAAGCCAGACCATACGAGTTCCCATGGCAGGTG
AY684711.1_1-842      CCCGGA---AAGATTGTCGGAGGAATTGAAGCCGGACCATACGAGTTCCCATGGCAGGTG
                                  ** ** ** ** ** *****  **************** *********

AB045720.1_1-845      TCCGTCCGAAGGAAGTCTTCCGATTCCCATTTCTGCGGAGGTAGCATCATCAACGATCGT
U25648.1_26-872       TCCGTCCGAAGGAAGTCTTCCGATTCCCATTTCTGCGGAGGTAGCATCATCAACGATCGT
AY438622.1_1-852      TCCGTCCGAAGGAAGTCTTCCGATTCCCATTTCTGCGGAGGTAGCATTATCAACGATCGT
EF545135.1_1-718      TCCGTCCGAAGGAAGTCTTCCGATTCCCATTTCTGCGGAGGTAGCATTATCAACGATCGT
U25643.2_20-869       TCCGTCCGAAGGAAGTCTTCCGATTCCCATTTCTGCGGAGGTAGCATCATCAACGATCGT
AF304199.1_1-850      TCCGTCCGAAGGAAGTCTTCCGATTCCCATTTCTGCGGAGGTAGCATCATCAACGATCGT
U25644.1_1-718        TCCGTCCGAAGGAAGTCTTCCGATTCCCATTTCTGCGGAGGTAGCATCATCAACGATCAT
AF433650.1_1-850      TCCGTCCGAAGGAAGTCTTCCGATTCCCATTTCTGCGGAGGTAGCATCATCAACGATCGT
AY684712.1_1-845      TCCGTCCGAAGGAAGTCTACCGATTCCCATTTCTGCGGAGGTAGCATCATAAACGATCGT
AY684711.1_1-842      TCCGTCCGAAGGAAGCCTTCTGATTCCCATTTCTGCGGAGGTAGCATCATCAACGATCGT
                      *************** ** * ************************** ** ******* *

AB045720.1_1-845      TGGGTTGTCTGCGCTGCTCACTGCATGCAGGGAGAGGCCCCCGCTCTGGTTTCATTGGTC
U25648.1_26-872       TGGGTTGTCTGCGCTGCTCACTGCATGCAGGGAGAGGCCCCCGCTCTGGTTTCATTGGTC
AY438622.1_1-852      TGGGTTGTCTGCGCTGCTCACTGCATGCAGGGAGAGAGCCCTGCCCTGGTTTCCTTGGTC
EF545135.1_1-718      TGGGTTGTCTGCGCTGCTCACTGCATGCAGGGAGAGAGCCCTGCCCTGGTTTCCTTGGTC
U25643.2_20-869       TGGGTTGTCTGCGCTGCTCACTGCATGCAGGGAGAGAGCCCTGCCCTGGTTTCATTGGTC
AF304199.1_1-850      TGGGTTGTCTGCGCTGCTCACTGCATGCAGGGAGAGAGCCCTGCCCTGGTTTCATTGGTC
U25644.1_1-718        TGGGTTGTCTGCGCTGCTCACTGCATGCAGGGAGAGAGCCCTGCCCTGGTTTCATTGGTC
AF433650.1_1-850      TGGGTTGTCTGCGCTGCTCACTGCATGCAGGGAGAGAGCCCTGCCCTGGTTTCATTGGTC
```

```
AY684712.1_1-845      TGGGTTGTCTGCGCTGCTCACTGCATGCAGGGAGAGAGCCCTGCCCTGGTTTCATTGGTC
AY684711.1_1-842      TGGGTTGTCTGCGCTGCTCACTGCATGCAGGGAGAGAGCCCTGCCCTGGTCTCATTGGTC
                      ***********************************   *** ** ***** ** ******

AB045720.1_1-845      GTGGGTGAGCACGACAGGAGTGCAGCGAGTACAGTACGTCAGACTCATGACGTTGATAGC
U25648.1_26-872       GTGGGTGAGCACGACAGGAGTGCAGCGAGTGCAGTACGTCAGACTCATGACGTTGATAGC
AY438622.1_1-852      GTCGGTGAGCACGACAGCAGCGCTGCGAGTACAGTACGTCAGACTCATGACGTTGACAGC
EF545135.1_1-718      GTCGGTGAGCACGACAGCAGCGCTGCGAGTACGGTACGTCAGACTCATGACGTTGACAGC
U25643.2_20-869       GTCGGTGAGCACGATAGCAGCGCTGCGAGTACAGTACGTCAGACTCATGACGTTGACAGC
AF304199.1_1-850      GTCGGTGAGCACGATAGCAGCGCTGCGAGTACAGTACGTCAGACTCATGACGTTGACAGC
U25644.1_1-718        GTCGGTGAGCACGATAGCAGCGCTGCGAGTACAGTACGTCAGACTCATGACGTTGACAGC
AF433650.1_1-850      GTCGGTGAGCACGATAGCAGCGCTGCGAGTACAGTACGTCAGACTCATGACGTTGACAGC
AY684712.1_1-845      GTCGGCGAGCACGATAGCAGCGCTGCGAGTACAGTTCGTCAGACTCATGATGTTGATAGC
AY684711.1_1-842      GTCGGCGAGCACGATAGCAGCGCTGCGAGTACAGTACGTCAGACTCATGATGTTGATAGC
                      ** ** ******** ** ** ** ****** * ** ************** ***** ***

AB045720.1_1-845      ATCTTCGTCCACGAGGACTACAACGCAAATACCCTAGAGAACGACGTTTCTGTCATCAAG
U25648.1_26-872       ATCTTCGTTCACGAGGACTACAACACAAATACCCTAGAGAACGACGTTTCTGTCATCAAG
AY438622.1_1-852      ATCTTCGTCCACGAGGACTACAACGGAAATACCTTTGAGAACGACGTTTCTGTCATCAAG
EF545135.1_1-718      ATCTTCGTCCACGAGGACTACAACGGAAATACCTTTGAGAACGACGTTTCTGTCATCAAG
U25643.2_20-869       ATCTTCGTCCACGAGGACTACAACGGAAATACCTTTGAGAACGACGTTTCTGTCATCAAG
AF304199.1_1-850      ATCTTCGTCCACGAGGACTACAACGGAAATACCTTTGAGAACGACGTTTCTGTCATCAAG
U25644.1_1-718        ATCTTCGTCCACGAGGACTACAACGGAAATACCTTTGAGAACGACGTTTCTGTCATCAAG
AF433650.1_1-850      ATCTTCGTCCACGAGGACTACAACGGAAATACCTTTGAGAACGACGTTTCTGTCATCAAG
AY684712.1_1-845      ATCTTCGTCAACGAAAACTACAATCCCCGTACACTTGAAAACGACGTTTCTGTCATCAAG
AY684711.1_1-842      ATCTTCGTCAACGAAAACTACGATCCCCGTACACTAGAAAACGACGTTTCTGTCATCAAG
                      ********  ****  ***** *      ***  * ** *********************

AB045720.1_1-845      ACATCTGTTGCCATCACTTTCGACATCAACGTTGGTCCAATCTGCGCCCCAGATCCGGCT
U25648.1_26-872       ACATCTGTTGCCATCACTTTCGACATCAACGTTGGTCCAATCTGTGCCCCAGATCCAGCT
AY438622.1_1-852      ACAGTTAACGCCATCGCTATCGACATCAACGTTGGGCCAATCTGCGCTCCAGATCCAGCC
EF545135.1_1-718      ACAGTTAACGCCATCGCTATCGACATCAACGTTGGGCCAATCTGCGCTCCAGATCCAGCC
U25643.2_20-869       ACAGTTAACGCCATCGCCATCGACATCAACGATGGGCCAATCTGCGCTCCAGATCCAGCC
AF304199.1_1-850      ACAGTTAACGCCATCGCCATCGACATCAACGATGGGCCAATCTGCGCTCCAGATCCAGCC
U25644.1_1-718        ACAGTTAACGCCATCGCCATCGACATCAACGTTGGGCCAATCTGCGCTCCAGATCCAGCC
AF433650.1_1-850      ACAGTTAACGCCATCGCCATCGACATCAACGTTGGGCCAATCTGCGCTCCAGATCCAGCC
AY684712.1_1-845      ACAGCTATCGCTATCACCTTCGACATTAACGTTGGGCCAATCTGTGCTCCAGATCCGGCT
AY684711.1_1-842      ACAGCTATCGCTATCACCTTCGACATCAACGTTGGACCAATCTGTGCTCCAGATCCGGCT
                      ***  *   ** *** *  ******* **** *** ******** ** ******** ** 

AB045720.1_1-845      AACGACTACGTCTACCGTAAGAGCCAGTGTTCCGGATGGGGAACTATCAATTCAGGTGGA
U25648.1_26-872       CAACAGTACGTCTACCGTAAGAGCCAGTGCTCCGGATGGGGAACTATCAATTCAGGTGGA
AY438622.1_1-852      AACGATTACGTCTACCGTAAGAGCCAGTGCTCCGGATGGGGAACTGTCAACTCAGGTGGA
EF545135.1_1-718      AACGATTACGTCTACCGTAAGAGCCAGTGCTCCGGATGGGGAACTGTCAACTCAGGTGGA
U25643.2_20-869       AACGATTACGTCTACCGTAAGAGCCAGTGCTCCGGATGGGGAACTATCAACTCAGGTGGA
AF304199.1_1-850      AACGATTACGTCTACCGTAAGAGCCAGTGCTCCGGATGGGGAACTATCAACTCAGGTGGA
U25644.1_1-718        AACGATTACGTCTACCGTAAGAGCCAGTGCTCCGGATGGGGAACTATCAACTCAGGTGGA
AF433650.1_1-850      AACGATTACGTCTACCGTAAGAGCCAGTGCTCCGGATGGGGAACTATCAACTCAGGTGGA
AY684712.1_1-845      AACGACTACGTCTACCGTAAGAGCCAGTGCTCTGGATGGGGATCTATAAACTCAGGTGGA
AY684711.1_1-842      AACGACTACGTCTACCGTAAGAGCCAGTGTTCCGGATGGGGAACTATCAATTCAGGTGGA
                       *  * *********************** ** ********* ** * ** *********

AB045720.1_1-845      ATCTGCTGTCCCAACGTTCTGCGATACGTGACGCTGAATGTCACAACCAACCAATTCTGC
U25648.1_26-872       ATCTGCTGTCCCAACATTCTGCGATACGTGACGCTGAATGTCACAACCAACCAATTCTGC
AY438622.1_1-852      GTCTGCTGCCCCAACGTTCTGCGATATGTGACACTGAACGTCACAACCAACGCCTTCTGC
EF545135.1_1-718      GTCTGCTGCCCCAACGTTCTGCGATATGTGACACTGAACGTCACAACCAACGCCTTCTGC
U25643.2_20-869       GTCTGCTGCCCCAACGTTCTGCGATATGTGACACTGAACGTCACAACCAACGCCTTCTGC
AF304199.1_1-850      GTCTGCTGCCCCAACGTTCTGCGATATGTGACACTGAACGTCACAACCAACGCCTTCTGC
U25644.1_1-718        GTCTGCTGCCCCAACGTTCTGCGATATGTGACACTGAACGTCACAACCAACGCCTTCTGC
AF433650.1_1-850      GTCTGCTGCCCCAACGTTCTGCGATATGTGACACTGAACGTCACAACCAACGCCTTCTGC
AY684712.1_1-845      ATCTGCTGTCCCGCAGTTTTGCGATATGTTACACTGAACATCACGACCAACGCCTTCTGC
AY684711.1_1-842      ATCTGCTGTCCCGCAGTTTTGCGATATGTTACACTGAACATCACGACCAACGCCTTCTGC
                       ******* ***    ** ******* ** ** *****  **** ******   ******
```

```
AB045720.1_1-845      GAAGATGTATAC---CCACTAAATTCAATCTACGACGATATGATTTGCGCGTCGGACAAC
U25648.1_26-872       GAAGATGTATAC---CCACTAAATTCAATCTTCGACGATATGATTTGCGCGTCGGACAAC
AY438622.1_1-852      GATGATATCTACAGCCCATTATATACAATTACCAGCGACATGATCTGCGCCACGGACAAC
EF545135.1_1-718      GATGATATCTACAGCCCATTATATACAATTACCAGCGACATGATCTGCGCCACGGACAAC
U25643.2_20-869       GATGATATCTACAGCCCATTATATACAATTACCAGCGACATGATCTGCGCCACGGACAAC
AF304199.1_1-850      GATGATATCTACAGCCCATTATATACAATTACCAGCGACATGATCTGCGCCACGGACAAC
U25644.1_1-718        GATGATATCTACAGCCCATTATATACAATTACCAGCGACATGATCTGCGCCACGGACAAC
AF433650.1_1-850      GATGATATCTACAGCCCATTATATACAATTACCAGCGACATGATCTGCGCCACGGACAAC
AY684712.1_1-845      GACGCCGTCTACA---CATCGGATACCATCTACGACGATATGATCTGCGCCACGGACAAC
AY684711.1_1-842      GACGCCGTCTACA---CATCGGACACTATCTACGACGATATGATCTGCGCCACAGACAAC
                      ** *   * ***    **    *  * **   *  *** ***** *****  * ******

AB045720.1_1-845      ACTGGGGGTAACGACAGAGACTCCTGCCAGGGTGACTCCGGCGGCCCTCTGAGCGTCAAG
U25648.1_26-872       ACTGGGGGTAACGACAGAGACTCCTGCCAGGGTGACTCCGGCGGCCCTCTGAGCGTCAAG
AY438622.1_1-852      ACCGGACAGAACGAGAGAGACTCTTGCCAGGGTGACTCTGGCGGCCCTCTGAGCGTCAAG
EF545135.1_1-718      ACCGGACAGAACGAGAGAGACTCTTGCCAGGGTGACTCTGGCGGCCCTCTGAGCGTCAAG
U25643.2_20-869       ACCGGACAGAACGAGAGAGACTCTTGCCAGGGTGACTCTGGCGGCCCTCTGAGCGTCAAG
AF304199.1_1-850      ACCGGACAGAACGAGAGAGACTCTTGCCAGGGTGACTCTGGCGGCCCTCTGAGCGTCAAG
U25644.1_1-718        ACCGGACAGAACGAGAGAGACTCTTGCCAGGGTGACTCTGGCGGCCCTCTGAGCGTCAAG
AF433650.1_1-850      ACCGGACAGAACGAGAGAGACTCTTGCCAGGGTGACTCTGGCGGCCCTCTGAGCGTCAAG
AY684712.1_1-845      ACTGGGATGACCGACAGAGACTCATGCCAGGGTGACTCCGGCGGCCCTCTGAGCGTCAAG
AY684711.1_1-842      ACTGGGATGACCGACAGAGACTCCTGCCAGGGTGACTCCGGCGGCCCTCTGAGCGTCAAG
                      ** **    * *** ******** ************** *********************

AB045720.1_1-845      GATGGCAGTGGAATCTTCAGCCTGATTGGTATTGTGTCTTGGGGAATTGGTTGCGCTTCT
U25648.1_26-872       GATGGCAGTGGAATCTTCAGCCTGATTGGTATTGTGTCTTGGGGAATTGGTTGCGCTTCT
AY438622.1_1-852      GATGGCAGCGGAATCTTCAGCCTCATTGGTATTGTGTCTTGGGGAATCGGTTGCGCATCT
EF545135.1_1-718      GATGGCAGCGGAATCTTCAGCCTCATTGGTATTGTGTCTTGGGGAATCGGTTGCGCATCT
U25643.2_20-869       GATGGCAGCGGAATCTTCAGCCTCATTGGTATTGTGTCTTGGGGAATCGGTTGCGCATCT
AF304199.1_1-850      GATGGCAACGGAATCTTCAGCCTCATTGGTATTGTGTCTTGGGGAATCGGTTGCGCATCT
U25644.1_1-718        GATGGCAGCGGAATCTTCAGCCTCATTGGTATTGTGTCTTGGGGAATCGGTTGCGCATCT
AF433650.1_1-850      GATGGCAGCGGAATCTTCAGCCTCATTGGTATTGTGTCTTGGGGAATCGGTTGCGCATCT
AY684712.1_1-845      GATGGCAGCGGAATCTTCAGTCTGGTTGGCATTGTGTCTTGGGGAATTGGTTGCGCTTCC
AY684711.1_1-842      GATGGCAGCGGAATCTTCAGTCTGGTTGGCATTGTGTCTTGGGGAATTGGTTGCGCCTCT
                      *******  *********** **  **** ***************** ******** **

AB045720.1_1-845      GGCTATCCAGGAGTCTACTCCCGCGTCGGATTCCATGCTGCATGGATCACCGACATCATC
U25648.1_26-872       GGCTATCCAGGAGTCTACTCCCGCGTCGGATTCCATACTGCATGGATCACCGACATCATC
AY438622.1_1-852      GGATATCCAGGAGTCTACGCCCGCGTCGGATCCCAAACTGGATGGATCACAGACATTATT
EF545135.1_1-718      GGCTATCCAGGAGTCTACGCCCGCGTCGGATCCCAAACTGGATGGATCACAGACATCATC
U25643.2_20-869       GGCTATCCAGGAGTCTACGCCCGCGTCGGATCCCAAACTGGATGGATCACAGACATTATT
AF304199.1_1-850      GGCTATCCAGGAGTCTACGCCCGCGTCGGATCCCAAACTGGATGGATCACAGACATTATT
U25644.1_1-718        GGCTATCCAGGAGTCTACGCCCGCGTCGGATCCCAAACTGGATGGATCACAGACATTATT
AF433650.1_1-850      GGATATCCAGGAGTCTACGCCCGCGTCGGGTCCCAAACTGGATGGATCACAGACATCATC
AY684712.1_1-845      GGCTATCCAGGAGTCTACTCCCGAGTCGGATTTCATGCTGGATGGATCACCGACACAATC
AY684711.1_1-842      GGCTATCCAGGAGTTTACTCCCGCGTCGGATTTCATGCTGGATGGATCACCGACACGATC
                      ** *********** *** **** ***** *  **   *** ********* ****  **

AB045720.1_1-845      ACCAACAACT--
U25648.1_26-872       ACCAACAACT--
AY438622.1_1-852      ACCAACAACTGA
EF545135.1_1-718      ACCAACAACT--
U25643.2_20-869       ACCAACAACT--
AF304199.1_1-850      ACCAACAACT--
U25644.1_1-718        ACCAACAACT--
AF433650.1_1-850      ACCAACAACT--
AY684712.1_1-845      ACCAACAACT--
AY684711.1_1-842      ACCAACAACT--
                      **********
```

---

clustalw.dnd

(
(
(
AY438622.1_1-852:0.00075,
EF545135.1_1-718:0.00482)
:0.00463,
(
AF433650.1_1-850:0.00023,
(
(
AB045720.1_1-845:0.00499,
U25648.1_26-872:0.01513)
:0.03733,
(
AY684712.1_1-845:0.01389,
AY684711.1_1-842:0.01699)
:0.04268)
:0.04439)
:0.00449)
:0.00255,
(
U25643.2_20-869:0.00000,
AF304199.1_1-850:0.00124)
:0.00040,
U25644.1_1-718:0.01224);

Select tree menu Exec
