# Supplementary material for: Cloning and enhancing lumbrokinase production from local Eisenia fetida by signal peptide engineering for effective thrombosis treatment
Source: PLoS One. 2025 Jul 24;20(7):e0328393. doi: 10.1371/journal.pone.0328393 (PMC12288994; doi:10.1371/journal.pone.0328393)
Supplement: S1 Table — (PDF) [file pone.0328393.s003.pdf]

S1 Table: Weight of clot after dissolution by incubating for different time intervals

Test Samples	30minutes	60minutes	120minutes	240minutes
PBS	0.283g±0.0040	0.283g±0.0041	0.283g±0.00405	0.283g±0.0042
0.5mg/ml Std. Lumbrokinase	0.275g ±0.0015	0.273g±0.0025	0.267g±0.00152	0.249g±0.00162
1mg/ml Std. Lumbrokinase	0.271g±0.00264	0.267g±0.00231	0.260g±0.00171	0.237g±0.002
0.5mg/ml recombinant Lumbrokinase	0.274g±0.00249	0.264g±0.0022	0.247g±0.00198	0.221g±0.0021
1mg/ml recombinant Lumbrokinase	0.267g±0.00233	0.258g±0.00211	0.241g±0.00233	0.184g±0.0026
1.5mg/ml recombinant Lumbrokinase	0.261g±0.0021	0.247g±0.00172	0.226g±0.00221	0.158g±0.0024
2mg/ml recombinant Lumbrokinase	0.241g±0.00214	0.226g±0.00198	0.191g±0.00243	0.1002g±0.0027

Weight of clot before dissolution = 0.298g
