# Supplementary material for: Cloning and enhancing lumbrokinase production from local Eisenia fetida by signal peptide engineering for effective thrombosis treatment
Source: PLoS One. 2025 Jul 24;20(7):e0328393. doi: 10.1371/journal.pone.0328393 (PMC12288994; doi:10.1371/journal.pone.0328393)
Supplement: S2 Table — (PDF) [file pone.0328393.s004.pdf]

S2 Table: Percentage clot lysis activity after different time of incubation.

**Test Samples**	**30minutes**	**60minutes**	**120minutes**	**240minutes**
PBS	5%	5%	5%	5%
0.5mg/ml Std. Lumbrokinase	7.80%	8.40%	10.30%	16.44%
1mg/ml Std. Lumbrokinase	8.90%	10.20%	12.59%	20.42%
0.5mg/ml recombinant Lumbrokinase	8.20%	11.30%	16.79%	25.67%
1mg/ml recombinant Lumbrokinase	10.30%	13.39%	18.83%	38.23%
1.5mg/ml recombinant Lumbrokinase	12.30%	16.80%	23.89%	46.78%
2mg/ml recombinant Lumbrokinase	18.90%	24.00%	35.67%	66.23%
